# The Identification of Drug Abuse

**DOI:** 10.3390/toxics11050444

**Published:** 2023-05-08

**Authors:** Maria Pieri

**Affiliations:** Department of Advanced Biomedical Science-Legal Medicine Section, University of Naples “Federico II”, Via S. Pansini, 5, 80131 Naples, Italy; maria.pieri@unina.it; Tel.: +39-0817463474; Fax: +39-0817464726

Forensic toxicology has played a central role since its development in defining mechanisms of acute intoxication, often with a lethal outcome. In its modern declination, the discipline has seen a constant evolution not only in the object of study—today extended to almost all psychoactive/psychotropic substances or any case capable of inducing morbid and/or toxic manifestations once absorbed by the organism—but also in its possible fields of application.

Analytical difficulties related to the need to highlight the presence of trace-level substances (i.e., a few nanograms or micrograms per mL) in complex biological samples are, in fact, only the first challenge in modern forensic toxicology. The constant improvement in analytical performance, linked to the optimization of purification procedures, the development of systems based on liquid or gaseous chromatography coupled to mass spectrometry, the availability of instruments that are capable of performing multiple scans of ionic signals of multiple substances within the same analytical run and the availability of constantly updated database libraries assist with the acquisition process of analytical data. The analytical aspect is, however, only the first part of the work required of a forensic toxicologist. Analytical purposes make it essential to correctly contextualize the analytical data in order to respond fully and exhaustively to the question posed by the Judicial Authority. The correct interpretation of analytical data is the most complex and debated aspect of forensic chemical-toxicological analysis. Difficulties derive from the need to integrate analytical results with evidence from all the other evaluations that the case under study requires.

Furthermore, the difficulties deriving from post-mortal modifications make the acquisition and correct interpretation of forensic toxicological analysis in the thanatological field profoundly different and significantly more complex than any analyses performed “on a living person”.

The availability of data in the literature obtained through validated analytical methods and results integrated with circumstantial, anamnestic and anatomopathological data represent a direction on which to develop a forensic chemical-toxicological analysis in line with the principles underlying the discipline, as well as legal requirements. Only a datum that has been characterized by analytical precision and correctly contextualized in the light of the main characteristics of the case under study is able to make a useful contribution to the resolution of the forensic problem that leads to an analytical request itself.

This Special Issue aims to present the analytical-interpretative difficulties that underlie the determination of different types of substances. The authors contribute with “real cases” and also address strategies through which it may be possible to resolve or at least contribute to the definition of possible causes of intoxication. Thirteen contributions (articles, case reports and reviews) are published.

Iqbali and Coll. present a UPLC-MS/MS method for the identification and quantification of lemborexant, a novel dual orexin receptor antagonist, that was recently approved for the treatment of insomnia [1]. Due to its potential for abuse, lemborexant is scheduled in the IV class by the United States Drug Enforcement Administration. The method presented here was previously validated according to the “Scientific Working Group for Toxicology” guidelines and subsequently cross-validated in rat plasma samples to be applied in pharmacokinetic studies.

Manetti and Coll. present a fatality involving an atypical transdermal patch consumption [2], starting from the results of a case report revealing the presence of fentanyl in a man with a history of illicit drug consumption. The authors review the literature on fatalities involving such synthetic opioids.

Carfora and Coll. present the case of a suicide involving helium, whose detection made it necessary for the development of adequate analytical procedures [3]. For an adequate sampling, the authors optimized the use of a special gas-inlet system provided with a vacuum, through which the sampled gas could be transferred to the mass spectrometer. 

Fernández-López and Coll. present a post-mortem study on the overdose caused by carbamazepine in a patient with psychiatric illness [4]. The analytical procedure to determine carbamazepine on human bone is presented and discussed.

Two articles focus on fatal pesticide outcomes, an occurrence still frequent in developing countries and also present in Western ones among agricultural workers. Simonelli and Coll. present a study on phorate ingestion that resulted in fatal outcomes [5]. Post-mortem data obtained from a 24-year-old Bengali male are described and commented on with respect to the literature. Basilicata and Coll. present the case of suicidal diquat ingestion in a 50-year-old man [6]. The subject was promptly rescued and transferred to the emergency department before they died the following day. The authors also focus also on the misconduct of sanitary staff during hospitalization, resulting in a decrease in the patient’s chances of survival and consequent professional liabilities.

In the studies of Hernandez and Coll. [7] and Albano and Coll. [8] the issue of prenatal drug exposure is presented. In the first study, the authors validate an analytical procedure to determine the main substance of abuse and metabolites in meconium [7]. Opioid absorption during intrauterine and prenatal life is then reviewed by Albano and Coll., presenting toxicological, clinical, and forensic issues [8].

Koželj and Prosen present data on the temperature-related degradation of tropanes atropine and scopolamine, highlighting the possible underestimation of GC/MS analysis in such tropane alkaloids as cases of the unintentional or intentional ingestion of plant material [9].

The review by Henríquez-Hernández and coll. discusses health-hazardous doses of psychedelic substances, with particular attention paid to ergolamines, simple tryptamines, and phenylethylamines [10].

Mannocchi and Coll. present their results on fatal intoxication related to acetaminophen, citalopram and trazodone [11]. Data from post-mortem analyses performed on the deceased found that an advanced state of decomposition was presented, which is commented on with respect to the available literature.

Scendoni and Coll. first report results for morphine determination in an unusual post-mortem matrix: in fingernails [12]. Data were first acquired by immunohistochemistry and subsequently confirmed by ultra-high-performance liquid chromatography coupled with high-resolution mass spectrometry.

In the paper from Basilicata and Coll., a case of incongruous midazolam administration in a terminal cancer patient is presented [13]. Toxicological data on different biological specimens are presented, and professional liabilities derived from non-adherence to guidelines on palliative care are discussed.

The items discussed in the present Special Issue highlight critical aspects of forensic toxicological activity with respect to both analytical and interpretative problems. Each contribution aimed to discuss a Special Issue related to drug misuse with respect to the most recent literature, thus representing a reference for those who are involved in the field.
